# Shear wave and strain sonoelastography for the evaluation of the Achilles tendon during isometric contractions

**DOI:** 10.1186/s13244-021-00974-y

**Published:** 2021-02-17

**Authors:** Alessandro Schneebeli, Ilaria Fiorina, Chandra Bortolotto, Marco Barbero, Deborah Falla, Corrado Cescon, Maria Vittoria Raciti, Francesco Tarantino, Lorenzo Preda

**Affiliations:** 1 Centre of Precision Rehabilitation for Spinal Pain (CPR Spine), School of Sport, Exercise and Rehabilitation Sciences, College of Life and Environmental Sciences, University of Birmingham, Birmingham, UK; 2 Rehabilitation Research Laboratory 2rLab, Department of Business Economics, Health and Social Care, University of Applied Sciences and Arts of Southern Switzerland, SUPSI, Manno/Landquart, Switzerland; 3 Dipartimento di radiologia, Fondazione IRCCS Policlinico San Matteo, Pavia, Italy

**Keywords:** Shear wave elastography, Strain elastography, Achilles tendon

## Abstract

**Objectives:**

Changes in mechanical loading as well as pathology can modify the Achilles tendon mechanical properties and therefore detection of these changes is relevant for the diagnosis and management of Achilles tendinopathy. The aim of this study was to evaluate strain and shear wave sonoelastography for their ability to detect changes in the Achilles tendon mechanical properties during a series of isometric contractions.

**Methods:**

Longitudinal sonoelastography images of the Achilles tendon were acquired from 20 healthy participants using four different ultrasound devices; two implementing strain sonoelastography technology (SE1, SE2) and two, shear wave elastography technology (SWE1, SWE2).

**Results:**

SE1 measured a decreasing strain ratio (tendon become harder) during the different contraction levels from 1.51 (0.92) to 0.33 (0.16) whereas SE2 mesaured a decreasing strain ratio from 1.08 (0.76) to 0.50 (0.32). SWE1 measured decreasing tendon stiffness during contractions of increasing intensity from 33.40 (19.61) to 16.19 (2.68) whereas SWE2 revealed increasing tendon stiffness between the first two contraction levels from 428.65 (131.5) kPa to 487.9 (121.5) kPa followed by decreasing stiffness for the higher contraction levels from 459.35 (113.48) kPa to 293.5 (91.18) kPa.

**Conclusions:**

Strain elastography used with a reference material was able to detect elasticity changes between the different contraction levels whereas shear wave elastography was less able to detect changes in Achilles tendon stiffness when under load. Inconsistent results between the two technologies should be further investigated.

**Supplementary Information:**

The online version contains supplementary material available at 10.1186/s13244-021-00974-y.

## Key points

- Achilles tendon disorders are common both in athletes and the general population.
- SWE and SE are ready to use tools to evaluate musculoskeletal structures.
- SE was able to detect elasticity changes between the different contraction levels.
- Inconsistency between SWE and SE should be further investigated.

## Background

Achilles tendon (AT) is the thickest tendon in the human body; it connects the triceps surae to the calcaneus bone and produces plantar flexion of the ankle. Injuries of the AT are often related to excessive loading during physical exercise [1]. Moreover, several mechanical and morphological changes of the AT occur with aging and in individuals with chronic tendinopathy [2, 3].

Ultrasonography evaluation is usually the first-line imaging examination for patients with suspected Achilles tendinopathy since it is widely available, has a relatively low cost and provides a real-time assessment [4]. Nevertheless, conventional ultrasound images can only provide information about the anatomical and morphological features of the AT and cannot assess tendon mechanical properties. More recently, AT stiffness and elasticity have been evaluated with different ultrasound-based technologies, in particular shear wave sonoelastography (SWE) and strain sonoelastography (SE) [5–10].

SE provides qualitative data of tissue elasticity on colour elastograms, exploiting the displacement determined by the operator through repeated compressions and de-compressions with the probe [9]. Given that the force applied by the operator to the tissue is unknown, in order to obtain a semi-quantitative evaluation of tissue elasticity, the ratio between two different region of interest (ROI) within the tissue is calculated. This “strain ratio” is often obtained by using subcutaneous fat [11] or external material [12, 13] as a reference tissue. In contrast, SWE applies an acoustic radiation force via the ultrasound beam to the tissue, in which the propagation velocity correlates with the tissue elasticity which can be quantified (in m/s or kPa) [6, 14].

Ultrasound-based elastography offers both researchers and clinicians a fast, non-invasive, quantitative assessment of tendon mechanical properties. It can be applied in the evaluation of acute and chronic Achilles tendinopathy [15, 16] or to define the response to several different mechanical loads [17] and additionally has the potential to improve the diagnosis of tendon disorders [18].

Although these technologies have been commonly used in research and clinical settings, the reliability of measurements from the AT is still debated [5, 12, 19, 20]. Limits and constraints of both SWE and SE have been reported [21] especially when used to analyse the tendon structure, indicating the importance of a systematic and structured approach. Nevertheless, some studies have confirmed the superiority of SE and SWE in terms of specificity, sensitivity and accuracy compared to conventional ultrasound for diagnosing Achilles tendinopathy [21]. Surprisingly, there has been no attempt to compare the available sonoelastography techniques. Therefore, the aim of this study was to evaluate and compare in vivo mechanical properties of the AT measured during contractions of increasing intensity when using three different SE/SWE devices. Additionally, the study aimed to establish if there is a correlation between the strain ratio methods and SWE in the evaluation of AT stiffness.

## Methods

Twenty asymptomatic volunteers (12 males, 8 females, mean age 28.95 ± 4.16 years) were recruited for this repeated measures study. Height (centimetres) and weight (kilograms) were recorded, and the body mass index (BMI) was calculated.

Subjects with a history of tendon injury and/or previous foot surgery or any painful episodes in the lower limbs in the last year, or systemic inflammatory disorders such as rheumatoid arthritis, spondyloarthropathies and hypercolesterolemia were excluded. All participants were informed about the content of the study and signed the informed consent form prior to the experimental procedure. The study was approved by the Ethics Committee of the Policlinico San Matteo, Pavia. (ID: 0579).

Longitudinal sonoelastography images of the left and right AT were performed with three ultrasound devices, using two different systems of SE and SWE technologies. The systems with SE were: Resona 7, Mindray, Shenzhen, China (SE1), and Aplio 500, Toshiba Medical Systems Corp., Tokyo, Japan (SE2). The systems with SWE were: Resona 7, Mindray, Shenzhen, China (SWE1), and Aixplorer; SuperSonic Imagine, Aix-En-Provence, France (SWE2).

**Fig. 1:**
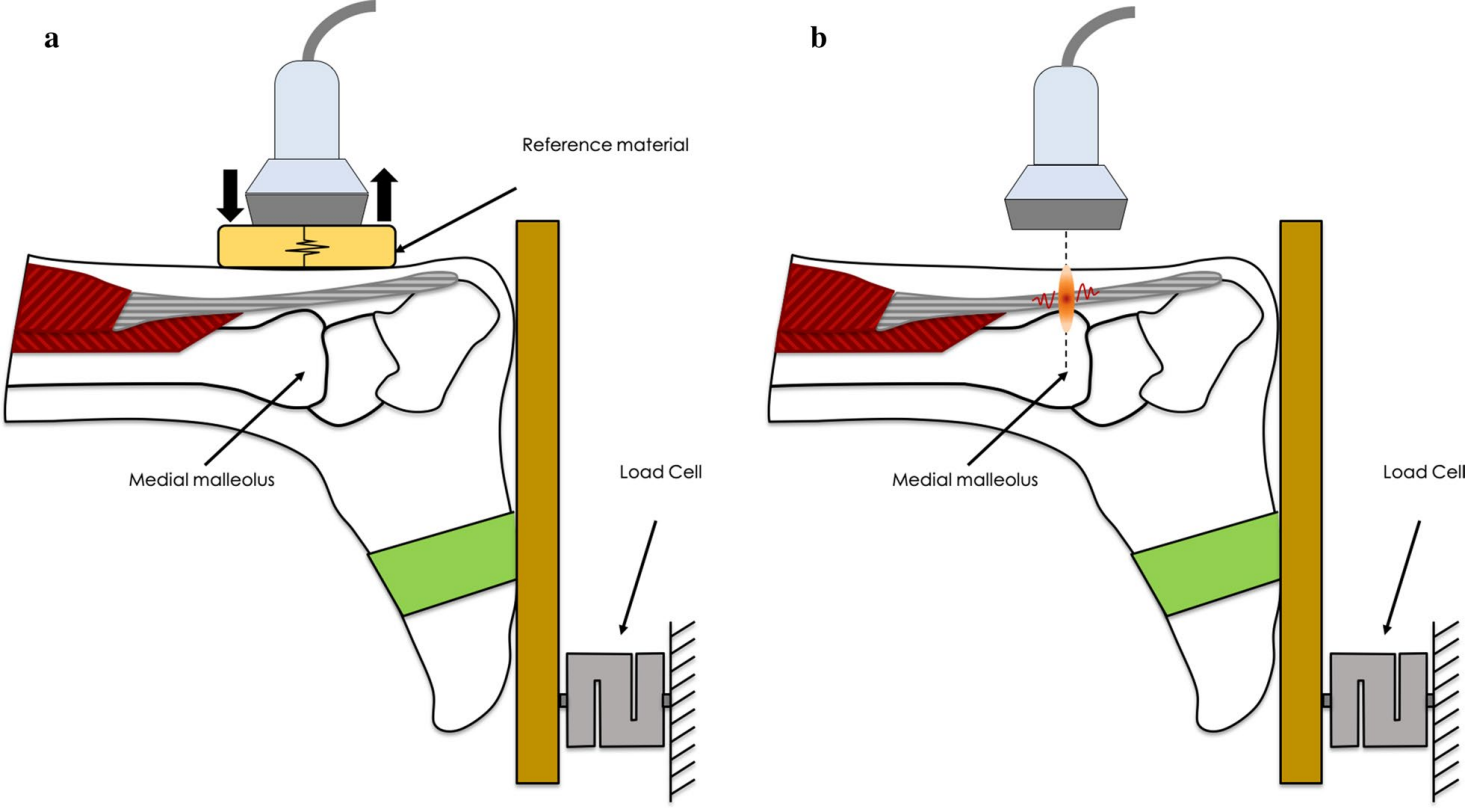
Schematic of the experimental setup. **a** Strain sonoelastography with reference material **b** Shear Wave sonoelastography. Ultrasound probe was placed in a longitudinal scan at the level of the medial malleolus

All examinations were performed bilaterally using multifrequency linear transducers (14L5 probe for Toshiba, L14-6VU for Mindray and 4–15 MHz, SL15-4 for Aixplorer). The participants were asked to lie in a prone position, exposing both AT, with the legs extended and the feet placed over the examination couch, in contact with a dynamometer (see Fig. 1).

The examination included AT long axis evaluation using B-mode, SE and SWE. Images were acquired at the level of the medial malleolus in order to minimize “bone proximity” hardening artefacts [22, 23]. The participants were instructed to perform contractions of different intensities (0 kg, 0.5 kg, 1 kg, 2 kg, 5 kg and 10 kg) in plantar flexion using an ankle ergometer; the examiner performed one measurement on each tendon at every load. The side of the tendon as well as the sequence of the isometric contractions was randomized.

All evaluations were performed by an experienced musculoskeletal radiologist with 7 years of experience with elastography and experience with all devices used.

### Data acquisition

Specific musculoskeletal presets were used and no ultrasound setting changes were allowed during the study; only simple tuning was allowed (e.g. depth, brightness and contrast) in order to obtain the best possible image. For the SE analysis, we used an external reference material (Zerdine®, CIRS, Inc., Norfolk) with known elastic properties (elasticity—102 kPa, speed of sound—1580 m/s, and attenuation coefficient—0.5 dB/cm/MHz). The external reference material was placed on the participant’s AT, included in the B-mode scans, and used to provide a comparison between the examined tendon and a material in which a constant elasticity was present.

SE images were analysed using a custom-made software; colour scale pixel count was made and colour histograms were extracted from two regions of interest (the reference material and AT). The range between soft and hard (from red to blue) was divided into 256 steps (0–255) according to the ultrasound image colour depth. Strain ratios between the tendon and the reference material were calculated and the comparison between different contraction levels were made. Higher strain ratio between the AT and the reference material indicates a softer tendon. This method has been validated, and reliability established in a previous study [12, 24]. The use of strain ratios with a reference material with known elasticity properties becomes even more relevant when comparing different ultrasound devices.

Shear wave images were analysed on the ultrasound device, and region of interest (ROI) were manually drawn in post-processing and included the full extent of the examined tendon displayable in the saved image. Reliability maps were available in dual screen for both systems and were used to check the quality of the obtained images. The acquired SWE-information was evaluated quantitatively; the shear elastic modulus (Young’s modulus) was calculated by the measuring tools of the ultrasound systems in kPa. This procedure was repeated for both AT and at different contraction levels. A higher value obtained with the SWE corresponds to a harder tendon. All images and data were stored in the ultrasound work-stations.

The comparison between different contraction levels for each of the ultrasound devices was made.

**Fig. 2:**
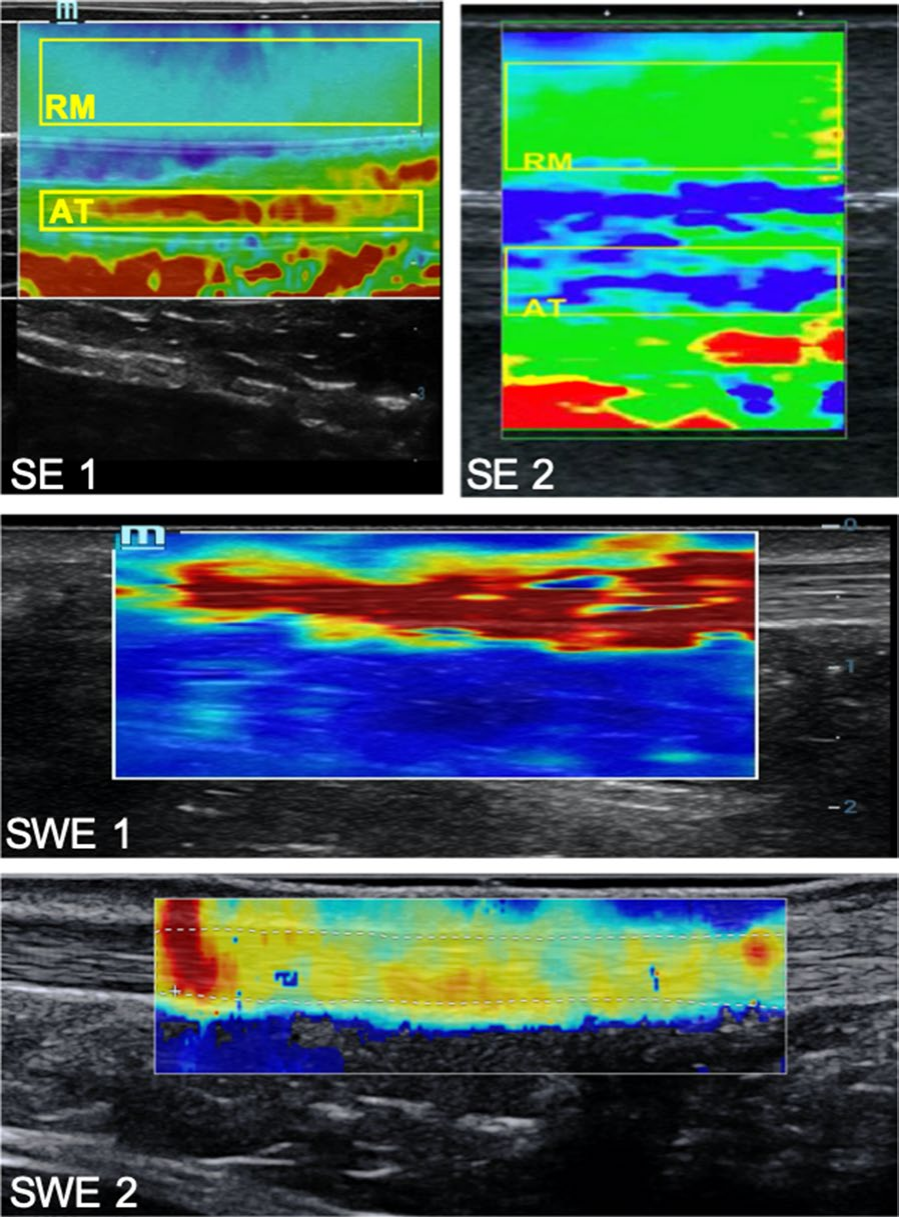
Example of the Achilles tendon mechanical properties at 0 kg measured with the different sonoelastography devices. SE1, strain elastography Resona 7, Mindray; SE2, strain elastography Aplio 500, Toshiba Medical Systems Corp.; SWE1, shear wave elastography Resona 7, Mindray; SWE2, shear wave elastography Aixplorer; SuperSonic Imagine, RM, reference material; AT, Achilles tendon

Figure 2 shows an example of SE and SWE images for the AT for the 0 kg load condition.

### Statistical analysis

The Shapiro–Wilk test was used to test the normal distribution of quantitative variables. Nonparametric, independent samples, Mann–Whitney test was used to assess differences in stiffness of the AT between the left and right side and between men and women.

A nonparametric test (Friedman test with post hoc pair wise comparison) was used to compare differences of stiffness for each of the ultrasound devices. Bonferroni correction was applied and an overall p value less than 0.05 was considered a significant difference. Medians and interquartile ranges (IQR 25th and 75th percentile) were used to report the results. Statistical analysis was performed using Statistical Package for the Social Sciences (SPSS) version 22.0 software (SPSS, Chicago, IL, USA).

## Results

There were no significant differences in elastographic parameters between genders or between the left and right AT, and therefore, the data were pooled and further analysed as an entire sample. The median age of the participants was 28 years (range 26–41 years), with a sex ratio (M:F) of 12:20. The mean ± SD height, weight and BMI were 173.6 (± 7.4) cm, 68.2 (± 10.5) kg and 22.5 (± 2.35), respectively.

### Comparison of the SE strain ratio values

**Fig. 3:**
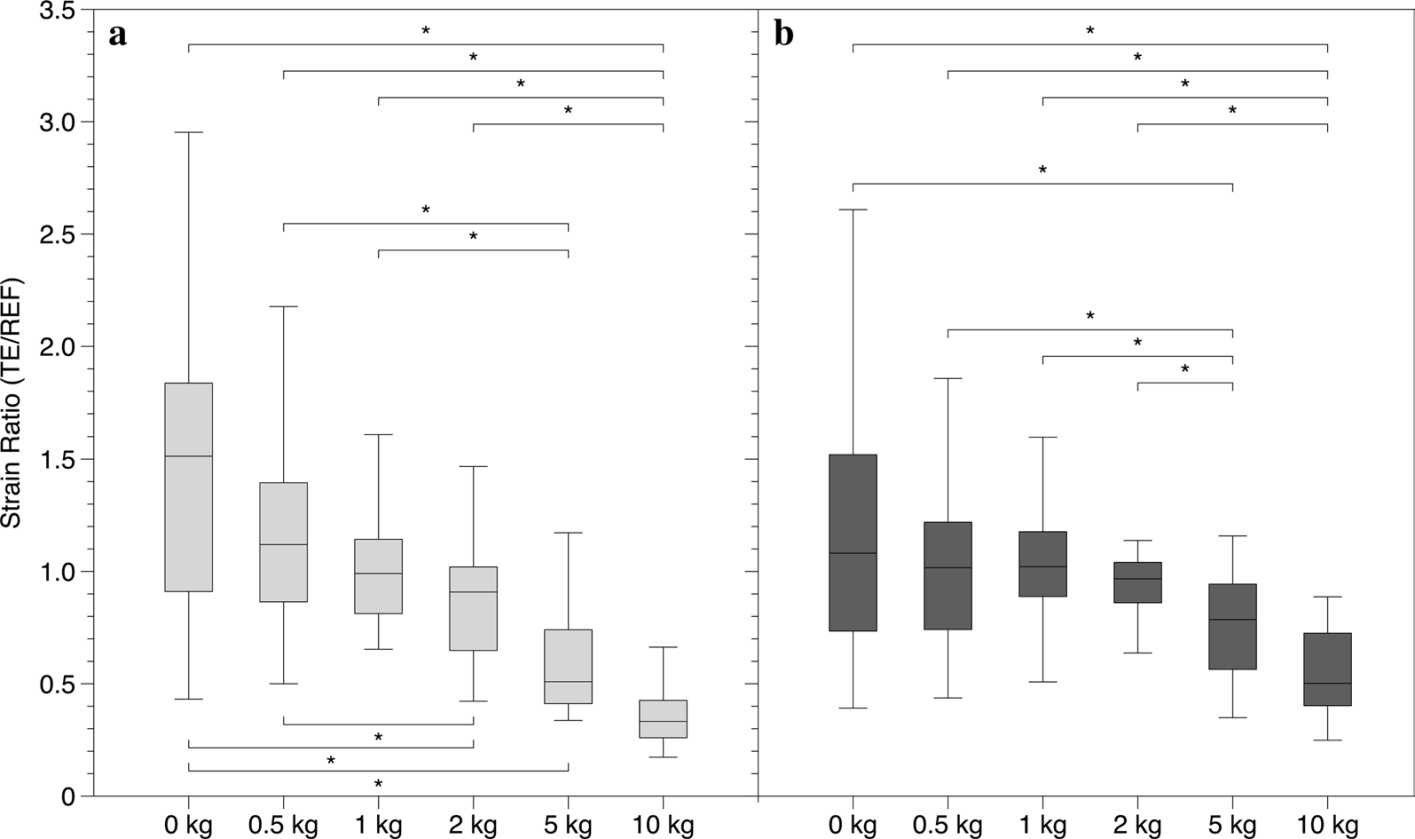
Strain sonoelastography. Box plot showing the median and IQR strain ratio (TE/REF) values of the entire sample for the different contraction levels **a** SE1 and **b** SE2. *p < 0.05; statistical significant difference between the different contraction levels

The median (IQR) values for the strain ratio measured at difference contraction levels are represented in Table 1. Evaluation with SE1 revealed a strain ratio of 1.51 (0.92) for the AT in the relaxed position and a progressive decrease of tendon strain ratio (the tendon became progressively harder) during increasing contraction levels, with median (IQR) values of 1.12 (0.49) at 0.5 kg, 0.99 (0.33) at 1 kg, 0.91 (0.37) at 2 kg, 0.50 (0.32) at 5 kg and 0.33 (0.16) at 10 kg. Friedman test for related samples revealed a significant difference (p < 0.01) between all contraction levels except for the adjacent levels. (Table 2 and Fig. 3a) Evaluation with SE2 revealed a strain ratio of 1.08 (0.76) for of the AT in the relaxed position, and a progressive decrease of the tendon strain ratio during contractions of increasing intensity, with median values of 1.02 (0.48) at 0.5 kg, 1.02 (0.26) at 1 kg, 0.97 (0.18) at 2 kg, 0.79 (0.37) at 5 kg, and 0.50 (0.32) at 10 kg. Friedman test for related samples, revealed a significant difference (*p* < 0.01) between the highest contraction levels (10 kg and 5 kg) and the other contractions but no difference (*p* > 0.05) between the adjacent and the lowest contraction levels. (Table 2 and Fig. 3b).

**Table 1:** Median (IQR) of the strain ratio (tendon/reference material) and Young’s modulus (kPa) at different contraction levels

	0 kg	0.5 kg	1 kg	2 kg	5 kg	10 kg
SE 1 (TE/REF)	1.51 (0.92)	1.12 (0.49)	0.99 (0.33)	0.91 (0.37)	0.50 (0.32)	0.33 (0.16)
SE 2 (TE/REF)	1.08 (0.76)	1.02 (0.48)	1.02 (0.26)	0.97 (0.18)	0.79 (0.37)	0.50 (0.32)
SWE 1 (kPa)	33.40 (19.61)	23.75 (10.12)	19.54 (6.48)	17.33 (4.41)	16.56 (4.09)	16.19 (2.68)
SWE 2 (kPa)	428.65 (131.5)	487.9 (121.5)	459.35 (113.48)	393 (83.83)	319.7 (94.75)	293.5 (91.18)

**Table 2:** Friedman test p values between the different contraction levels for the different technologies

SE2	SE1						SWE2	SWE1					
	**0 kg**	**0.5 kg**	**1 kg**	**2 kg**	**5 kg**	**10 kg**		**0 kg**	**0.5 kg**	**1 kg**	**2 kg**	**5 kg**	**10 kg**
0 kg	–	1.000	0.405	**0.000**	**0.000**	**0.000**	0 kg	–	0.073	**0.000**	**0.000**	**0.000**	**0.000**
0.5 kg	1.000	–	1.000	**0.002**	**0.000**	**0.000**	0.5 kg	0.625	–	0.475	**0.011**	**0.000**	**0.000**
1 kg	1.000	1.000	–	0.632	**0.000**	**0.000**	1 kg	1.000	1.000	–	1.000	0.127	0.106
2 kg	1.000	1.000	1.000	–	0.062	**0.000**	2 kg	1.000	0.053	0.735	–	1.000	1.000
5 kg	**0.004**	**0.001**	**0.000**	**0.015**	–	0.062	5 kg	**0.000**	**0.000**	**0.000**	**0.008**	–	1.000
10 kg	**0.000**	**0.000**	**0.000**	**0.000**	0.128	–	10 kg	**0.000**	**0.000**	**0.000**	**0.001**	1.000	–

Bold values indicate statistically significant difference between the different contraction levels *p* < 0.05

### Comparison of the SWE Young’s modulus (kPa)

**Fig. 4:**
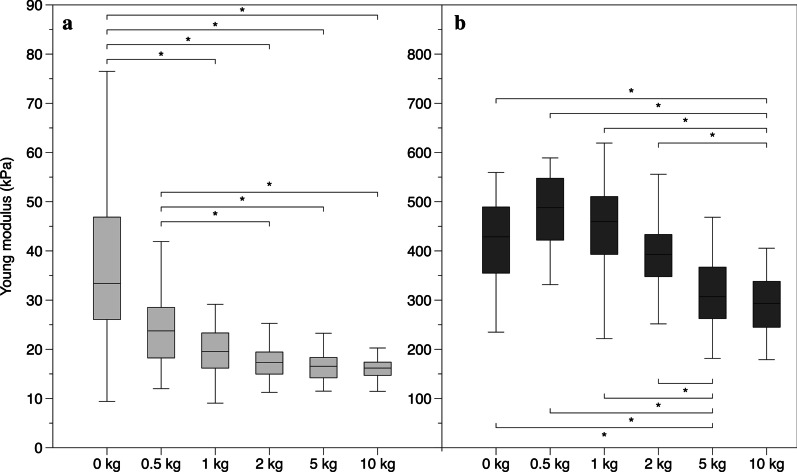
Shear wave sonoelastography. Box plot showing the median Young’s modulus and IQR values of the entire sample for the different contraction levels **a** SWE1 and **b** SWE2. *p < 0.05; statistical significant difference between the different contraction levels

Evaluation with SWE1 revealed a median (IQR) elastic modulus of 33.40 (19.61) kPa for the AT in the relaxed position and a progressive decrease of Young’s modulus (indicating reduced stiffness) during the progressive contraction levels, with median values of 23.75 (10.12) kPa at 0.5 kg, 19.54 (6.48) kPa at 1 kg, 17.33 (4.41) kPa at 2 kg, 16.56 (4.09) kPa at 5 kg and 16.19 (2.68) kPa at 10 kg. Friedman test for related samples revealed a significant difference (*p* < 0.01) between the lowest contraction levels (0 kg and 0.5 kg) and the other contractions but no difference (*p* > 0.05) between the adjacent and the highest contraction levels. (Fig. 4a).

Evaluation with SWE2 showed median (IQR) elastic modulus of 428.65 (131.5) kPa for the AT in the relaxed position and an increase of Young’s modulus (higher stiffness) to 487.9 (121.5) kPa at 0.5 kg followed by a progressive decrease of tendon modulus (reduced stiffness) during higher contraction levels, with median values of 459.35 (113.48) kPa at 1 kg, 393 (83.83) kPa at 2 kg, 319.7 (94.75) kPa at 5 kg and 293.5 (91.18) kPa at 10 kg. Friedman test for related samples revealed a significant difference (*p* < 0.01) between the highest contraction levels (10 kg and 5 kg) and the other contractions but no difference (*p* > 0.05) between the adjacent and the lowest contraction levels. (Fig. 4b).

## Discussion

This study was undertaken to assess the consistency of SE and SWE in the evaluation of the AT at different contraction levels, to compare the elasticity results using commercially available scanners and to explore the performances using different ultrasound imaging. Different behaviour was observed for the two sonoelastography techniques as well as for the different devices indicating limitations of both techniques.

The observed differences can be explained by the inconsistent methodological and computational approaches used to estimate tissue stiffness/elasticity. This has important consequences for both researchers and clinicians. Secondary research such as systematic reviews or meta-analysis is also hindered due to the limitations when pooling datasets from different studies. The lack of a reference standard constitutes a barrier for a diffuse application of SE/SWE the clinical practice.

The behaviour of both SE1 and SE2 is similar to previously published studies showing a decrease of the strain ratio values when the tendon becomes harder due to an increase in plantarflexion strength [24] or with supraspinatus contraction [25] and similar results were obtained using a semi-quantitative method with SE [26]. Moreover, these results are in line with a myotonometry evaluation of the AT [27] which showed increased stiffness when the tendon is under load.

As shown by Schneebeli et al. [24], higher variability of strain ratio values between the participants was present in the relaxed position and at low levels of contraction. This variability is reduced when the tendon is put under load. This behaviour is clearly visible in both SE techniques (Fig. 3) and could be explained by inter-individual difference in age, level of activity and anatomical differences (gastrocnemius and soleus length). A lack of accuracy of these techniques in the relaxed position, leading to a large variability in the strain ratio results, cannot be excluded. However, this method has high intra-rater reliability when used in both a relaxed and contracted state [12].

Strain ratio values between SE1 and SE2 appear to be different with SE1, showing a relaxed value of 1.51 (0.92) and a rapid decrease in strain ratio when the tendon is put under load (see Fig. 3), while SE2 showed a starting value of 1.08 (0.76) and similar values at the low contraction levels. Values of SE1 are highly comparable with another study [24] reporting a strain ratio of 1.61 (1.5–2.9) in the relaxed state, 1.30 (1.07–2.02) at 0.5 kg, 1.00 (0.76–1.66) at 1 kg, 0.81 (0.70–1.19) at 2 kg, 0.47 (0.39–0.73) at 5 kg and 0.33 (0.28–0.40) at 10 kg. Pairwise comparison between different contraction levels showed that SE1 could not detect changes between contraction levels of similar intensity and at the beginning of the isometric contraction (0–0.5 kg (*p* = 1.000), 0–1 kg (*p* = 0.405)). Similarly, SE2 could not distinguish the adjacent contraction levels but additionally, differences between low level contraction levels were not detected by this device. Differences were only observed at the higher contraction levels (5 kg, 10 kg). This low sensitivity in the initial phase of the contraction could be attributed to a different colour scale implemented in the device that considers mid-range values (green levels) more so than the higher or lower values.

Conversely, SWE using two different devices showed a different result compared to SE and to the expected biomechanical behaviour. Previous studies evaluating AT at different ankle angles indicate an increase in the shear modulus and shear wave velocity with an increase in ankle dorsiflexion [5, 8, 19, 28–31].

In this study, SWE 1 showed the opposite behaviour with a decrease in shear modulus with an increase in the contraction level and thus tension on the AT. It could be that the upper limit of the device (66.7 kPa) was surpassed. The implemented algorithm in SWE 1 does not present saturated values as a loss of signal (as for SWE 2) (Additional file 1: Appendix 1).

Moreover, the values obtained with SWE 1 at 0° of ankle plantar flexion (33.40 (19.61)) are extremely low compared to values reported in other studies [5, 32, 33], questioning the possibility of using Mindray shear wave sonoelastography to analyse tendon mechanical properties.

Values reported in Table 2 and Fig. 4a, which indicate a significant difference between different contraction levels, should be interpreted cautiously given the unusual shear modulus values indicating an opposite behaviour of this technology compared to SE and biomechanical studies. Further studies using SWE 1 are needed to evaluate the feasibility of this technology to measure mechanical properties of tendinous structures.

SWE 2 measured an increase of tendon stiffness during isometric plantar flexion from 0 to 0.5 kg load with shear modulus median values ranging from 428.65 (131.5) to 487.9 (121.5). This increment is followed by a progressive decrease of the Young’s modulus for isometric plantar flexion at 1, 2, 5 and 10 kg (see Table 1). This behaviour does not reflect tendon biomechanics, and it appears that the upper limit of the device (800 kPa) was reached. SWE 2 images of isometric contraction higher than 0.5 kg revealed signal saturation with a signal loss within the elastogram. This signal loss is probably due to the alignment or the displacement of the fibrillary structure within the AT that could increase the speed of the shear waves or could generate an abnormal propagation of the shear wave that cannot be registered by the device.

The absence of signal within some portion of the AT could have led to a miscalculation of the shear modulus resulting in a reduction of the kPa which was not attributed to the actual behaviour of the tendon. (Additional file 1Appendix 2). Given the upper limit of the shear modulus in these technologies, some authors suggested [5, 32] to limit the evaluation of AT to a relaxed or plantar flexed position and to not put tendon under load by means of a dorsiflexed position or any additional weight.

SWE has been largely used to evaluate the AT, a in natural relaxed position. In 1165 healthy Chinese adults, a Young’s modulus of 374.24 ± 106.12 kPa was reported [31]. However, inconsistency between studies exists when tendon is analysed at 0° of plantar flexion as in the present study. Shear modulus varied from 723.1 (kPa) [5] to 174.1 (kPa) [19] with one study reporting 322 (kPa) for the dominant leg and 320 (kPa) for the non-dominant [33]. Chino et al. [32] reported values to those measured with SWE2 in the present study with 430 (± 96) kPa at measured at 10% of the lower limb length and 470 (± 151) kPa at 15% of the lower limb length. The present study reported a median (IQR) value of 428.65 (131.5).

Even though these studies evaluated AT in the free tendon part, differences between results could be attributed to the transducer position along the free tendon or to challenges using this technology to evaluate the tendon under tension as in the 0° ankle position.

Using SWE 1, we measured median (IQR) values of 33.40 (19.61) at 0° of plantar flexion. Since this device, to our knowledge, has never been used to analyse tendinous structures, there are no data to compare to. However, considering the values obtained with other devices, these results appear to be far below the expected kPa values questioning the possibility to compare the results between different ultrasound devices.

The lack of relationship between these two methods may be related to the inherent differences in the parameters measured. SE evaluates tissue elasticity before and after manual compression, while SWE estimates Young’s modulus on the basis of the ultrasound propagation shear wave velocity. In addition, SWE is more susceptible to measurement interference due to the anisotropic nature of the tendon.

Considering the fundamental role of load in the development of tendon pathology as well as in the management of tendon rehabilitation, by means of loaded-based exercises, a device which is able to detect changes in the mechanical properties of tendon during tasks that directly challenge the tendinous structure (i.e. contraction) could provide a better understanding of tendon injury and rehabilitation effects. Strain evaluation appears to be the best technique to assess the elastic changes of the AT during isometric plantar flexion, nevertheless SE provides only semi-quantitative values (strain ratios) and this data, unlike Young’s modulus, does not represent the tissue intrinsic properties [21]. Moreover, the use of manual compression and reference material required higher technical skills leading to a more operator-dependent procedure.

There are some limitations to the current study. First, the selection of the devices and technology used was limited to those available. Second, the application of SE and SWE was limited to the evaluation of the AT. Generalizability of these findings to other devices and other tendons may not be possible. Further studies, which also evaluate the mechanical properties of pathological tendons, are necessary.

## Conclusion

SE with the use of reference material is able to detect changes in AT elasticity when measured in ankle plantar flexion during different contraction levels, and the results are in line with the expected physiological and mechanical behaviour of the tendon. Nevertheless, SE can only provide semi-quantitative (i.e. strain ratio) values and requires higher technical skills for operation. Conversely, SWE measured changes in tendon stiffness which were contradictory to the mechanical characteristics of the AT, probably due to the propagation shear wave velocity and the fibrillary structure of the AT. SE appears more suitable for the evaluation of the AT when under load. Caution should be taken when comparing the results of different devices  used to assess the AT. The inconsistency of the results between the different devices may hinder the clinical applicability of SE and SWE.

## Supplementary Information

**Additional file 1.** 1. Example of SWE 1 measurement during isometric contraction from 0 kg to 10 kg. Red values correspond to higher stiffness whereas blue values represent lower stiffness. 2. Example of SWE 2 measurement during isometric contraction from 0 kg to 10 kg. Red values correspond to higher stiffness whereas blue values represent lower stiffness. Signal loss in the Region of Interest are visible after 0.5 kg of isometric contraction.

## Data Availability

The datasets used and/or analysed during the current study are available from the corresponding author on reasonable request.

## Abbreviations

AT: Achilles tendon
SE: Strain elastography
SWE: Shear wave elastography
ROI: Region of interest
IQR: Interquartile ranges
