# Supplementary material for: Shear wave and strain sonoelastography for the evaluation of the Achilles tendon during isometric contractions
Source: Insights Imaging. 2021 Feb 17;12:26. doi: 10.1186/s13244-021-00974-y (PMC7889779; doi:10.1186/s13244-021-00974-y)

**ELECTRONIC SUPPLEMENTARY MATERIAL**

Appendix

1. Example of SWE 1 measurement during isometric contraction from 0kg to 10kg.

Red values correspond to higher stiffness whereas blue values represent lower stiffness.


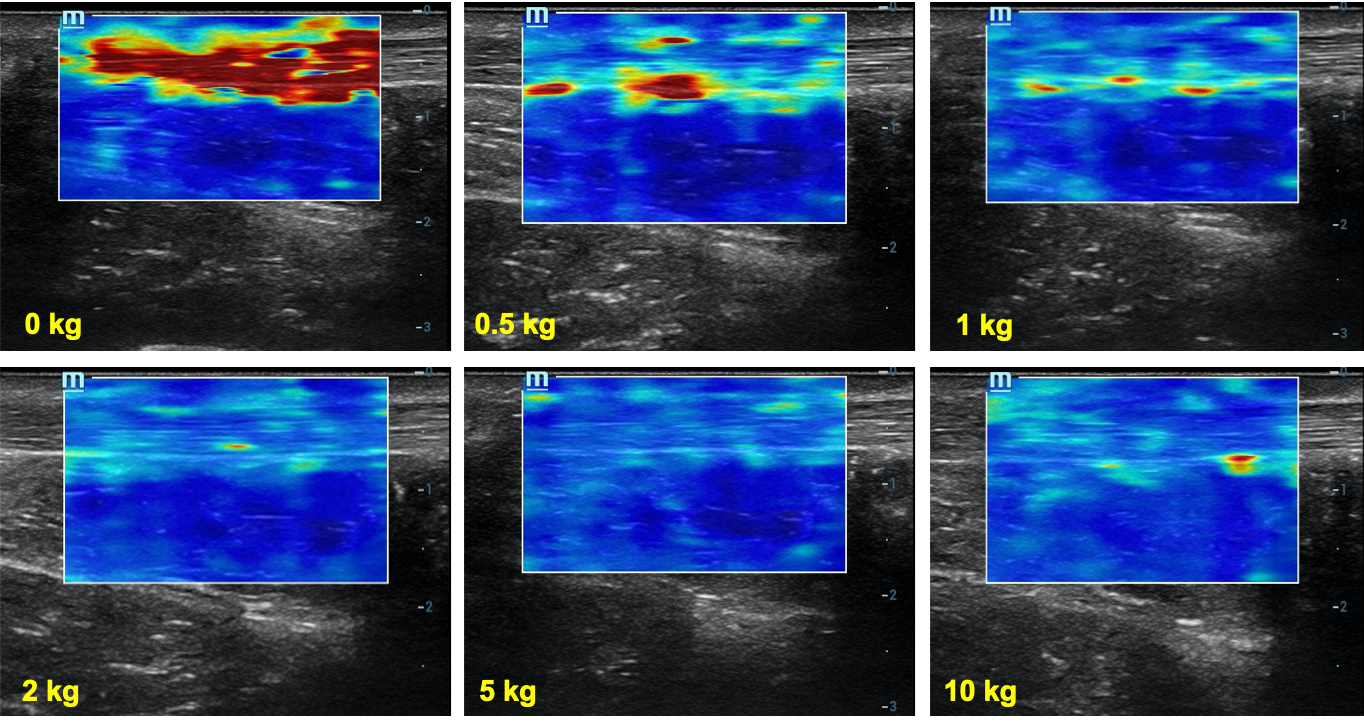


2. Example of SWE 2 measurement during isometric contraction from 0kg to 10kg.

Red values correspond to higher stiffness whereas blue values represent lower stiffness.

Signal loss in the Region of Interest are visible after 0.5 kg of isometric contraction.


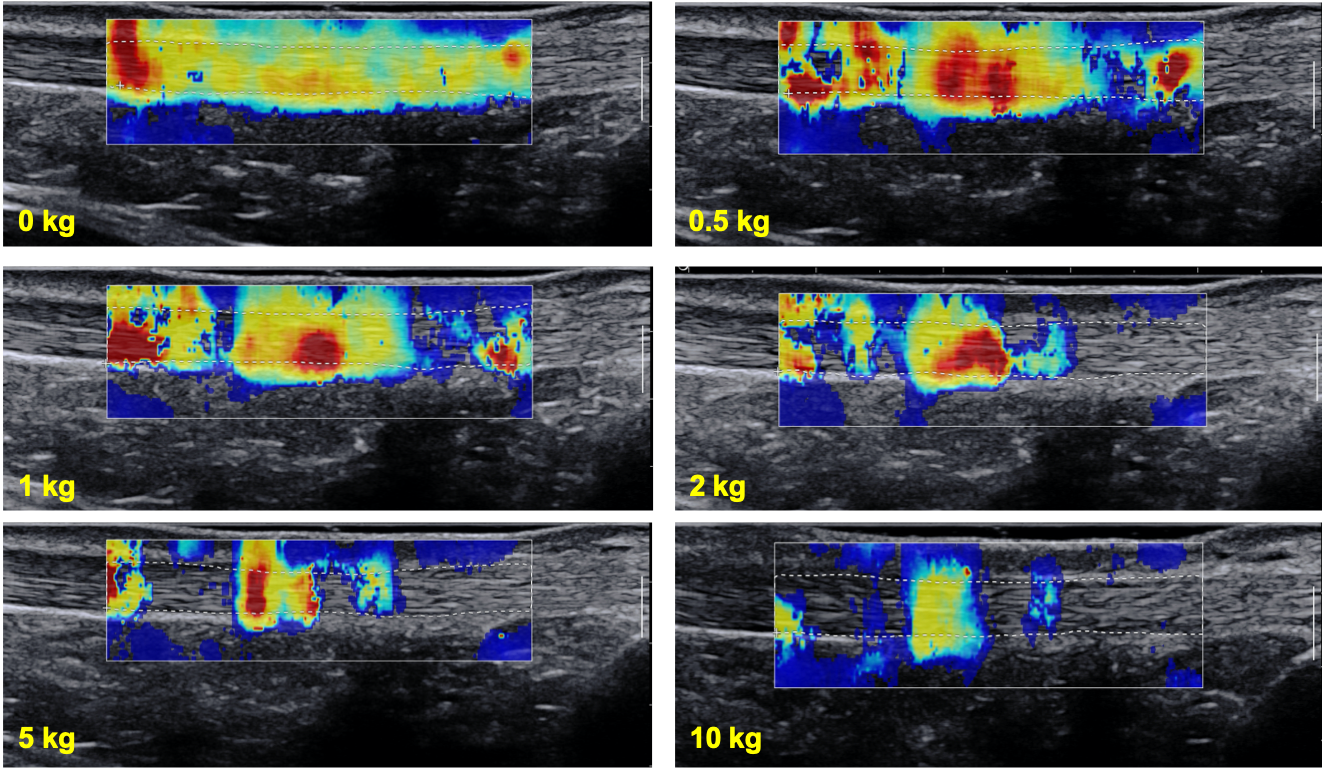

Supplement: Supplementary file 1 — Additional file 1. 1. Example of SWE 1 measurement during isometric contraction from 0 kg to 10 kg. Red values correspond to higher stiffness whereas blue values represent lower stiffness. 2. Example of SWE 2 measurement during isometric contraction from 0 kg to 10 kg. Red values correspond to higher stiffness whereas blue values represent lower stiffness. Signal loss in the Region of Interest are visible after 0.5 kg of isometric contraction. [file 13244_2021_974_MOESM1_ESM.docx]
